# Attention costs drive differences between active and passive risk-taking

**DOI:** 10.1038/s41598-026-43415-w

**Published:** 2026-05-13

**Authors:** Christian König-Kersting, Johannes Lohse, Anna Louisa Merkel

**Affiliations:** 1 Jönköping International Business School, Jönköping University, Jönköping, Sweden; 2 Institute for Economics, Leuphana University, Lüneburg, Germany; 3 Alfred-Weber-Institute for Economics, Heidelberg University, Heidelberg, Germany; 4 Department of Economics, University of Birmingham, Birmingham, UK

**Keywords:** Risk-taking, Mode-of-choice, Passive decision making, Attention costs, Human behaviour, Reward

## Abstract

Humans change their exposure to risks either actively, by taking deliberate actions, or passively, through inaction. These different modes of choice may result in varying levels of risk exposure. This study introduces a novel experimental task, the Dynamic Lottery Adjustment Task (DLAT), to investigate the effects of active versus passive risk-taking across two related experiments. The DLAT addresses a gap in the literature, as existing incentivized risk measures exclusively target active risk-taking. Consequently, differences between active and passive risk-taking have previously been studied only through non-incentivized surveys and vignettes focusing on specific choice domains (e.g., vaccination decisions). The first study, a controlled laboratory experiment, shows little variation in risk-taking between active and passive choice modes, contradicting existing findings of a general “passive-is-less-risky” bias. Conversely, the second experiment, conducted online over ten days, introduces higher attention costs and provides strong evidence that these costs significantly influence risk-taking behaviors in more naturalistic decision-making environments. Our findings highlight the importance of considering situational factors, such as attention costs, in understanding how different choice modes affect risk-taking in real-life settings, including financial investments, health behaviors, or career choices.

## Introduction

Risk-taking is a fundamental aspect of human decision-making across various fields, including economics, finance, psychology, medicine, sports, crime, and cybersecurity^1–8^. Intuitively, we associate the term “risk-taking” with a deliberate decision to take a risky action, such as engaging in a dangerous sport or betting on an unlikely event. However, an equally important but often overlooked aspect involves inaction — for example, failing to engage in preventive behaviors like health screenings or financial planning^9,10^. Despite this potentially important distinction, most of the common decision-theoretic models of risk-taking, such as expected utility theory^11,12^ and prospect theory^13^, focus on expected outcomes rather than the mode of choice, whether active (risk resulting from taking an action) or passive (risk resulting from not taking an action)^14–20^. Consequently, experiments in economics and psychology typically employ tasks that require participants to actively select a preferred lottery or risk level^21–30^. In this paper, we introduce a novel experimental design that allows to explore the differences between active and passive risk-taking, presenting findings from two studies utilizing this design to explore whether there is a general “passive-is-less-risky” bias.

The mode of risk-taking, whether active or passive, may influence risk exposure in distinct ways. For instance, problematic levels of active risk-taking, such as gambling or substance abuse, have been associated with personality traits like sensation seeking^31,32^ or impulsivity^33^, often reflecting a strong preference for immediate hedonic rewards over potential future losses. Conversely, passive risk-taking, which involves inaction, is likely linked to personality traits that inhibit action^34^. At a motivational level, it may also present a specific case of a broader class of inaction phenomena like omission bias^35,36^ and status quo bias.^37,38^ For example, individuals may experience less regret from negative outcomes resulting from passive risks compared to actively pursued ones. Additionally, inaction requires less effort and attention than taking action, making it less susceptible to distractions or attention costs^39^. In line with these considerations, surveys measures have identified active and passive risk-taking as distinct risk domains, each associated with different personality traits and supported by specific situational factors^34,40–42^.

A key claim in this literature is that passively accepted risks are perceived as less risky than equivalent actively taken risks i.e. a “passive-is-less-risky” bias^34^. Consequently, passive risk-taking may lead to greater overall risk-taking compared to active risk-taking. However, this claim is primarily supported by surveys that rely on self-reported behaviors or comparisons of active and passive risk scenarios from different domains^40,41^. Instead, a behavioral measure that ensures comparability across active and passive decision modes, while keeping other relevant aspects such as risk and return constant, remains absent from the literature. Such a measure would clarify whether a general “passive-is-less-risky” bias exists^34^ or if reported differences are due to specific contextual features of the vignette scenarios used, such as the timing of consequences or the particular domains studied (e.g., vaccination decisions or cybersecurity risks). More broadly, a context free behavioural measure would also assess whether standard risk elicitation procedures, which focus almost exclusively on active risk-taking, adequately capture all aspects of risk behavior.

There are different possible ways to think about the phenomena of active and passive risk-taking. Our perspective is that individuals are constantly exposed to unrealized risks in their daily lives and this background *risk exposure* can be affected through different actions and underlying mechanisms. One useful lens is whether changes in risk exposure are implemented through *commission* (doing something) or *omission* (not doing something), often described as opt-in versus opt-out choice architectures. A large behavioral literature suggests that acts and omissions can be experienced differently, in part because anticipated regret and perceived responsibility may be more salient when outcomes can be attributed to an active choice, as emphasized in regret-based accounts of decision making under uncertainty^43^ and in classic discussions of omission bias in vaccination decisions^35^. At the same time, outside the laboratory, commission and omission are often confounded with differences in the underlying source of risk, for example vaccinating replaces disease risk with a qualitatively different, typically smaller, side-effect risk, which makes it difficult to separate mode-of-choice effects from domain-specific perceptions of risk.

Against this background, we distinguish two stylized environments in which risk exposure can evolve. First, in some situations, an individual’s risk exposure in a given domain (e.g. financial or health) remains unchanged by default and increases or decreases only through taking a deliberate action, for example purchasing stocks instead of holding cash. We term this the *no-change* environment. In contrast, in other settings risk exposure changes by default, and deliberate action may be required to limit or amplify exposure. For example, such a *change* environment can arise when an individual holds a stock portfolio without regularly re-balancing it, such that risk exposure changes over time even in the absence of active trading. In our experiment, a natural interpretation of the ACTIVE and PASSIVE implementations is that they vary whether movement along an otherwise identical adjustment path is driven by an act or by inaction, while holding the risk domain and the set of available lotteries constant. This feature is intended to reduce confounds or interpretive difficulties that commonly arise in field settings, and it provides a structured way to study whether, and in which direction, the mode of choice is associated with differences in chosen risk exposure.

So far little is known how individuals change their exposure to risk if they are in a change or a no-change environment. We address this gap in the existing literature and attempt to capture the main dynamics of changes to individuals’ risk exposure with the Dynamic Lottery Adjustment Task (DLAT). In this paper we introduce the DLAT as a new experimental task in which participants actively increase or decrease their exposure to risk or passively allow changes in risk exposure to take place until taking an action. After outlining the general methodology, we present two experiments using the DLAT to explore how different modes of choice affect monetary risk-taking.

The first experiment is a laboratory study employing a two-by-two design, varying the mode of choice (active/passive) and the initial lottery endowment (risky/safe). This setup isolates the effects of the mode of choice when action or inaction increase or decrease risk exposure. The second experiment is conducted online with decisions being made over a span of ten days. This extended timeframe enhances attention costs and resembles more closely natural risk-taking environments, such as those in personal investment decisions. In this context, we think of attention as an effortful directed action rather than an involuntary reaction. Directing attention to a particular task requires effort and therefore comes at a cognitive cost. Such costs include remembering to take action as well as doing the steps required to take action such as logging into an online interface. This costly attention perspective is in line with traditional psychological interpretations of attention and effort as well as economists’ idea of attention as a scarce cognitive resource^39,44,45^. Our second experiment therefore allows us to investigate attention costs as a potentially significant situational driver of passive risk-taking.

Results from Experiment 1 show no significant differences in risk-taking due to the mode of choice, regardless of whether choices increase or decrease risk exposure relative to an initial endowment. Rather, participants initially endowed with a safe lottery took less risk than those endowed with a risky lottery, independent of the mode of choice. In contrast, Experiment 2 reveals substantial mode-of-choice effects: participants took more risks when inaction led to greater risk and less risk when inaction led to smaller risks.

In sum, this paper makes two key contributions: First, we introduce an experimental task for studying active and passive risk-taking that can be flexibly adapted to various research questions. Second, systematically covering all possible combinations of decision mode (active or passive) and choice outcome (increase or decrease exposure), we demonstrate that there is no general “passive-is-less-risky” bias as suggested by prior findings. Consequently, an explanation based on differential regret from outcomes resulting from passive rather than active risk-taking holds little traction in a task that controls for all other situational factors. However, when we reintroduce attention costs as a plausibly important factor differentiating passive and active choice situations, we observe differences in risks taken actively and passively. Thus, rather than through a perception bias or differential regret, passive risk-taking may result from a failure to initiate preventative measures when attention costs are high. Similarly, risk-avoidance is more frequent when implemented automatically than when requiring action. These findings suggests that existing active behavioral measures of risk-taking used in experimental work may well overlook some critical aspects of risk-taking when inaction changes risk exposure.

## Methods

### Participants

Both studies were conducted at the AWI Lab at Heidelberg University using hroot for participant recruitment^46^ and oTree for programming and running the experiment^47^. We investigated the phenomenon in a standard student sample and in a traditional laboratory setting that guaranteed maximum control over the decision-situation and minimized outside influences. Based on a conventional alpha level of 0.05 and a power $(1-\beta )$ of 0.8, and an initially targeted sample size of 100 per treatment group for the ACTIVE vs. PASSIVE comparison, we were powered to detect effect sizes of about 0.41, i.e., effects of small to medium size. This translates to differences between active and passive modes of about 1.05 lottery adjustment steps or a difference in the coefficient of relative risk aversion of 0.47 given the observed sample mean and assuming equal standard deviations for both treatments. Taking the starting points (SAFE/RISKY) into account halves the sample size to 50 per group comparison. At the same power and alpha levels, we were sufficiently powered to detect medium to large effects (Cohen’s d = 0.58), which translates into differences in 1.29 lottery adjustment steps or a difference in the coefficient of relative risk aversion of 0.62.

In Experiment 1, 199 participants (SAFE/ACTIVE: 52, SAFE/PASSIVE: 52, RISKY/ACTIVE: 48, RISKY/PASSIVE: 47) took part, with each session lasting approximately 45 min. The sample consisted of 80% native German speakers, 57% females, and 28% economics students, with an average age of 22.9 years. Participants received a fixed show-up fee of EUR 3.00 and a variable payment depending on their choices in the experiment as well as random draws to determine the payoff-relevant lotteries. Average earnings were EUR 9.87, including a EUR 3.00 show-up fee. In Experiment 2, 212 participants (SAFE/ACTIVE: 47, SAFE/PASSIVE: 59, RISKY/ACTIVE: 54, RISKY/PASSIVE: 52) took part in the initial session, which lasted no more than 20 min. Further decisions were made online in the 10 days subsequent to the initial session. The sample included 86% native German speakers, 57% females, and 26% economics students, with an average age of 23.6 years. Choices in Experiment 2 were incentivized like in Experiment 1. Including the show-up fee, participants earned an average of EUR 10.50.

### The dynamic lottery adjustment task

We propose the DLAT as a new risk elicitation procedure in which participants change their exposure to risk either through taking action or through remaining passive. We base this procedure on the Ordered Lottery Selection (OrdLS) procedure,^21,48^ adapting both the presentation of lotteries and the selection mechanism. The traditional OrdLS task involves actively selecting one lottery from a set, with each lottery offering two equally likely outcomes. The expected payoffs range from safe (both outcomes yield the same payoff) to very risky (one outcome yields no and the other outcome a very high payoff), with increasing expected payoff and variance moving from safe to risky. In the DLAT, instead of presenting all lotteries simultaneously for active selection, we present one lottery at a time. This lottery represents a participant’s current risk exposure. Participants then decide whether to retain this lottery and associated risk exposure or adjust its payoffs, thereby moving to a new lottery with new payoffs. If they choose to adjust, the process continues until they either retain the current lottery or reach the maximum number of adjustment steps. The construction of lottery payoffs and additional aspects of the task are detailed in the Supplementary Information (SI: Section A). Full instructions are provided in SI: Section D. Information regarding replication packages and data can be found at the end of this document.

**Fig. 1:**
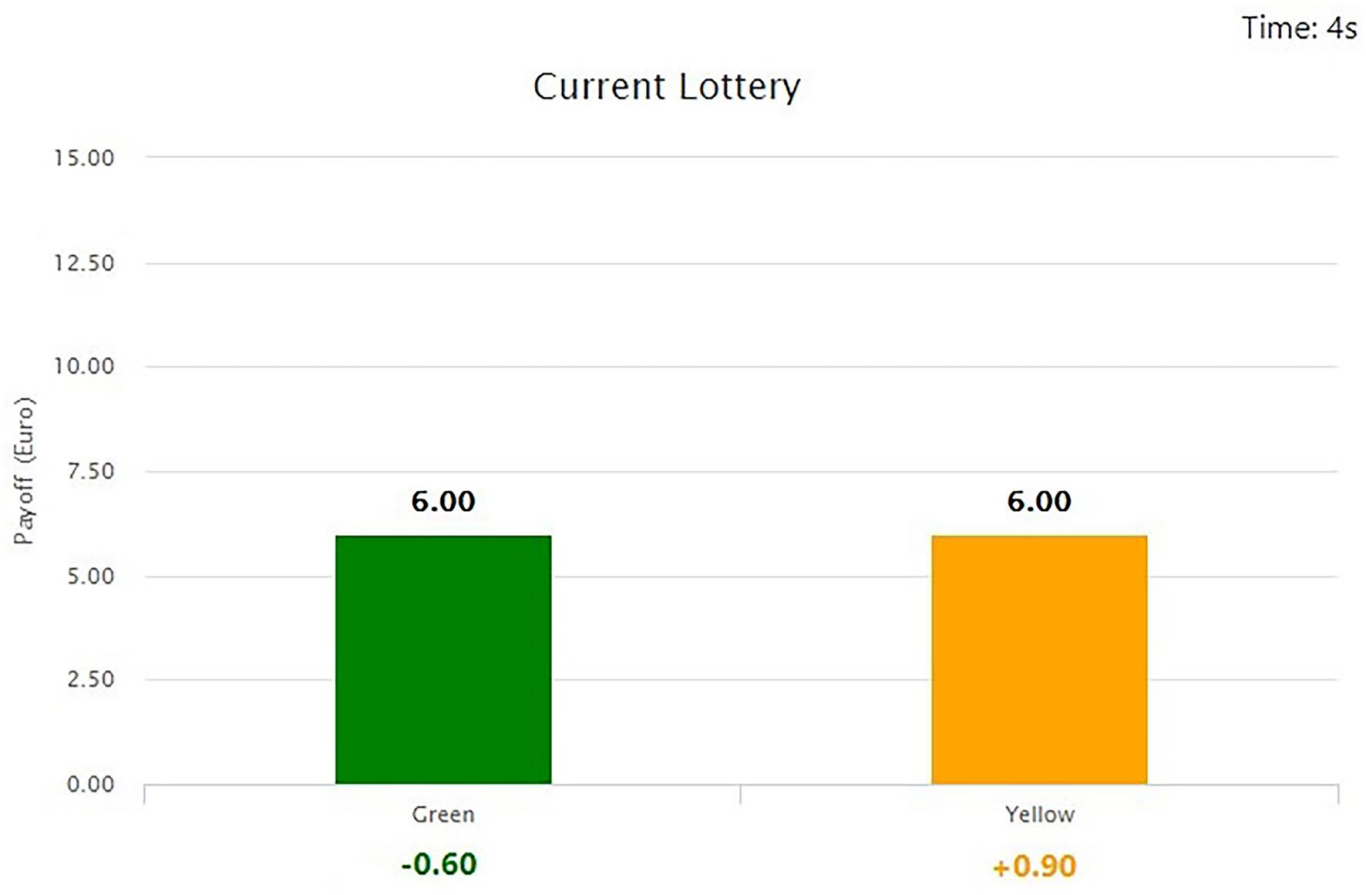
Example decision screen. This Figure represents lottery F1.5-Medium at step $s=0$. All values are in Euros.

On participants’ decision screens, lotteries are displayed as bar charts. Each bar represents the payoff associated with one of the two possible outcomes, labeled as Green and Yellow. The number below each bar indicates the change in payoffs that would result if participants choose to adjust their current lottery. For example, in Fig. 1 the current lottery is a safe lottery that exposes the participants to no risk as they receive EUR 6.00 in each possible outcome. The next adjustment step would result in more risk exposure by moving to a new lottery yielding EUR 5.40 (− 0.60) if the outcome is Green and EUR 6.90 (+ 0.90) if the outcome is Yellow. Participants can cycle through up to ten adjustment steps, with each step leading to the same change in payoffs for the Green and Yellow outcomes.

We chose this task format to implement two treatment dimensions. To test for mode-of-choice effects, lottery adjustment steps can be implemented through action or inaction. In the ACTIVE condition, participants must click a button within a fixed period to adjust lottery payoffs; if they do not, the current lottery is selected. In the PASSIVE condition, lottery payoffs adjust automatically at the end of each period unless participants click a button to stop the process. If they do not click the button, adjustments continue up to ten times. Thus, in the ACTIVE treatment, each adjustment step (and associated change in risk exposure) requires action, while in the PASSIVE treatment, adjustments (and associated changes to risk exposure) occur automatically unless interrupted by the participant. Importantly, all other features of the decision process, except for the way in which changes to risk exposure are implemented, are constant across the two decision modes. Specifically, the adjustment process occurs at the same intervals in both the ACTIVE and PASSIVE conditions. In the ACTIVE condition, adjustments are not immediate but happen after the same time has passed as in the PASSIVE condition. Immediate adjustments in the ACTIVE condition would make conditions less comparable, as participants could cycle through multiple adjustments more quickly.

Second, to determine if mode-of-choice effects depend on whether adjustments lead to more or less risk-taking, participants start with either a SAFE or RISKY initial lottery. In the SAFE condition, participants begin with a safe lottery that pays the same amount for each outcome. Each adjustment step decreases the Green outcome’s payoff and increases the Yellow outcome’s payoff by a larger amount, increasing both expected payoffs and variance (i.e., increasing reward and risk exposure). In the RISKY condition, participants start with the riskiest lottery, where only the Yellow outcome yields a positive payoff. Each adjustment step increases the Green outcome’s payoff and decreases the Yellow outcome’s payoff by a larger amount, reducing both variance and expected value (i.e., decreasing risk exposure and reward).

Apart from allowing for a direct comparison of active and passive decision modes, there are three further advantages of the DLAT over existing behavioral tasks and survey measures: First, the DLAT is an abstract lottery selection task without any contextual framing, avoiding context-rich environments that may interact with choice modes or distract from the main risk-reward trade-off. This makes it ideal for studying the general effect of different choice modes on risk-taking, rather than their effects in specific contexts like vaccination decisions. Second, the DLAT’s design allows for studying mode-of-choice effects in settings where adjustments lead to either more or less risk-taking. In this abstract setting, participants can be endowed with either a RISKY or SAFE starting lottery without changing instructions or other task aspects. Third, the DLAT is linear in probabilities, making it easy to visually illustrate that expected payoffs and their variance increase or decrease with each adjustment step^24,25^. More complex, non-linear tasks are arguably harder for participants to understand and tend to generate more noise and inconsistent choices.^29,49^

### Design and procedures of Experiment 1

We implement a two-by-two between-subject design, varying both the mode of choice (ACTIVE/PASSIVE) and the initial lottery endowment (SAFE/RISKY). There are ten distinct decision rounds of the DLAT, with participants remaining in the same treatment throughout. The rounds differ in lottery payoffs and adjustment step sizes, allowing us to test if mode-of-choice effects depend on stake and adjustment step size. The expected payoff from the riskiest lottery varies from EUR 2 to EUR 18 across the ten rounds.

The rounds are divided into two blocks of five. In the first block, adjustments to the Yellow outcome are 1.5 times larger than those to the Green outcome (Factor 1.5 lotteries). In the second block, the adjustment factor is 3 (Factor 3 lotteries). We counterbalance the presentation order of the blocks; some participants encounter Factor 1.5 lotteries first, while others start with Factor 3 lotteries. Within each block, lotteries occur in a fixed, non-monotonic order to prevent participants from anticipating the payoff changes that could occur in subsequent lottery rounds. That is, we always present the lottery with the medium stake size and the the mediums sized adjustment steps first, before alternating between larger stakes and larger step size adjustments. In the SAFE treatments, for example, the order of Factor 1.5 rounds and adjustment step sizes is: (− 0.6, + 0.9), (− 1.0, + 1.5), (− 0.2, + 0.3), (− 0.8, + 1.2), and finally (− 0.4, + 0.6). Note that the 10 adjustment steps in each lottery round have the same size. That is, in the first Factor 1.5 lottery round, each adjustment step changed the payoffs of the lottery by − 0.6 and + 0.9 for the Green and Yellow outcomes respectively. The time between individual payoff adjustments is set to 5 s in all lottery rounds. To familiarize participants with the decision screen and the adjustment process, there is a trial period before the first decision is made (SI: Fig. S1 in Section B). Additionally, participants have 15 s at the start of each round (i.e., for each new lottery round) to observe the round specific parameters (i.e., starting values and adjustment step size). Participants do not receive any feedback on lottery outcomes for a given round until the end of the experiment to prevent conditioning choices on past results or payments. SI: Tables S1 and S2 (Section C), provide details on all lotteries and payoffs. Following the main tasks, we collect additional information on the experiment’s content, participants’ demographics, and stated and revealed risk preferences.

### Design and procedures of Experiment 2

The key difference between Experiment 1 (lab) and Experiment 2 (online) is the time gap between adjustment steps. In the lab, lottery adjustments occur every five seconds, so participants need to pay attention for at most 50s across ten adjustment steps. This short time-span keeps attention and effort costs relatively low and constant across the ACTIVE and PASSIVE conditions. While this design feature allows for a clean experimental comparison between both conditions, it abstracts from the effort, attention, and opportunity costs of active decision-making. To reintroduce these features, Experiment 2 is conducted online over ten days, with each adjustment step occurring approximately 24h apart.

There is a single DLAT round, with each adjustment step occurring one day apart. Each day, participants either actively click a button (ACTIVE) or refrain from action (PASSIVE) to make adjustment decisions. We use the same two-by-two design as in Experiment 1, where adjustments result in either higher (SAFE) or lower (RISKY) risk exposure.

To facilitate the longer decision horizon, we created a website for participants to make choices remotely. Adjustments are approximately equally spaced, with participants submitting decisions within a four-hour window each day, which they chose before the experiment began. This design ensures that failures to make timely decisions are not due to pre-existing commitments. The selected time window remains constant throughout the experiment.

Participants can only make decisions during their chosen time window, and outside of this window cannot see the current lottery or make choices. Just like in Experiment 1, decisions to stop adjustments (PASSIVE) or to refrain from triggering another adjustment (ACTIVE) are final. Participants in the PASSIVE condition cannot restart adjustments after stopping, and those in the ACTIVE condition cannot skip a day and continue to make further adjustments the next day.

In Experiment 2, the initial and final lottery payoffs match the Factor 1.5-Medium lottery from Experiment 1, but exact adjustment step sizes vary around a midpoint daily and are revealed only after they occur. This variation prevents participants from inferring the exact number of steps needed to reach their preferred lottery and therefore from bypassing daily attention and effort costs in either the ACTIVE or PASSIVE conditions (see SI: Section A). As a result, the active and passive conditions now differ sharply in the action and attention required to implement a preferred level of risk. Active choices require daily attention to adjust risk to the preferred level, whereas passive choices require attention only to halt the automatic adjustment process at the right time.

Experiment 2 spans three phases. On day 1, participants attended a briefing session in the lab, where they received instructions, asked questions, provided demographic information, created a unique participation code, selected a four-hour decision window, and were assigned to either the ACTIVE or PASSIVE condition, receiving a EUR 3.00 show-up fee. From day 2, participants made online adjustment decisions over ten days using their unique code, which linked their choices to their demographic data and was used for payment. On day 12, they completed an online questionnaire for an additional EUR 2.00, and then received their lottery earnings and other payments via bank transfer on the following business day.

## Results

### Data analysis

In both studies, we use the coefficient of constant relative risk aversion (r) implied by participants’ lottery choices as our main measure of risk preferences. Assuming a constant relative risk aversion (CRRA) utility function ($u\left( x\right) =\frac{x^{1-r}}{1-r}$) , participants’ lottery choices translate into a range of possible r values consistent with these choices. A higher r coefficient implies more aversion to risk. In line with existing literature, we use the midpoints of these ranges as the implied risk aversion coefficient. For those who select either the first or the last lottery, we use the upper or lower bound of the interval as the measure of implied risk aversion. SI Table S3 (Section C) summarizes the implied risk aversion coefficient ranges and their midpoints for the different lotteries used in the experiment. We report complementary findings for a simpler measure (the number of lottery adjustments) in SI: Tables S4 and S5 (Section C). Our main results are robust to using either measure. For all statistical analysis we report results of non-parametric (Mann–Whitney U tests, Sign Rank Test) and parametric (Multivariate Regression) tests.

### Experiment 1

**Fig. 2:**
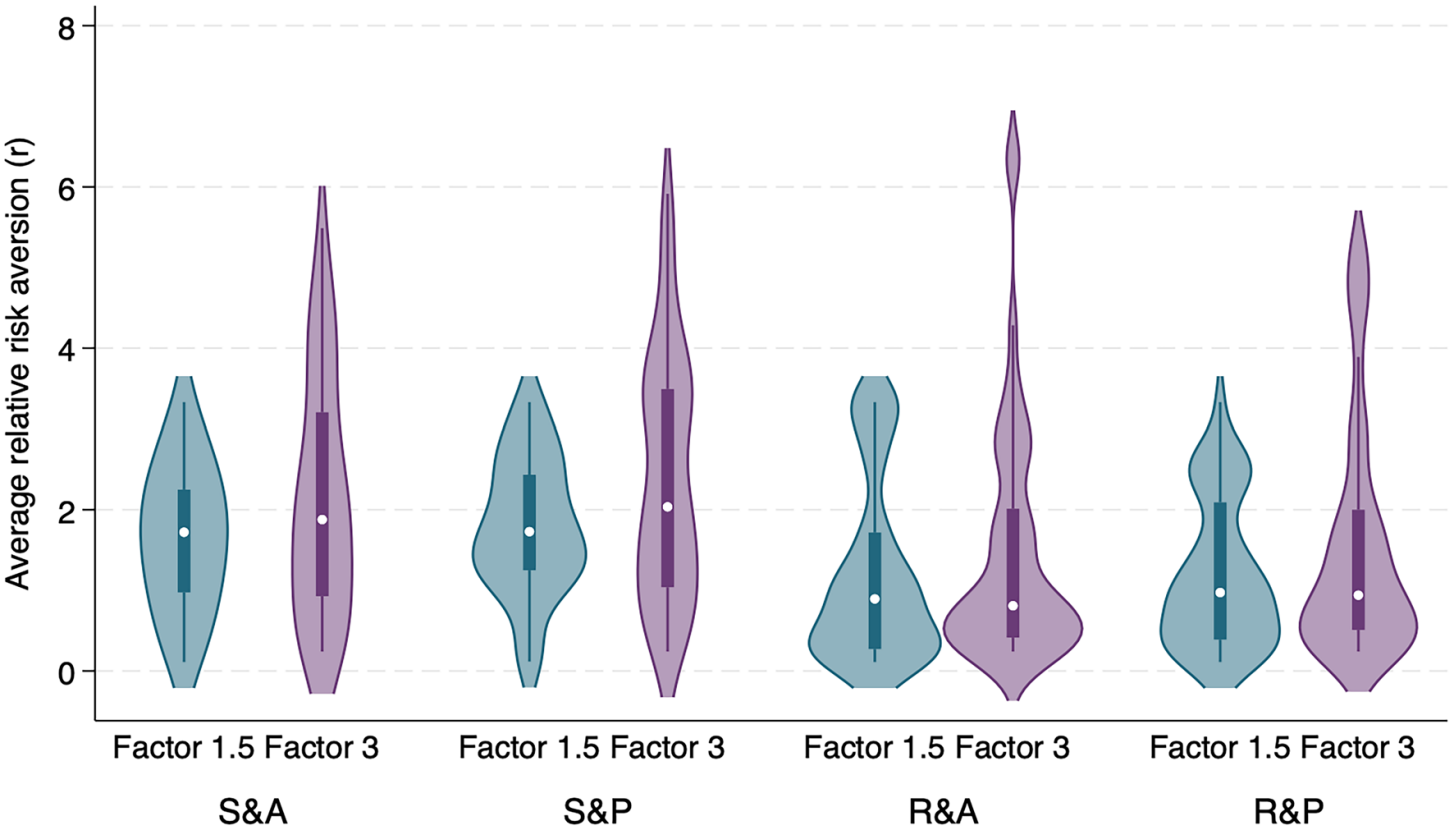
Experiment 1: Risk aversion by treatment. The figure shows an overlay of violin and box plots of relative risk aversion coefficients (r) by treatment. Coefficients are averaged over all lottery decisions by treatment condition, differentiated by Factor 1.5 and Factor 3 lotteries.

We first investigate whether a mode-of-choice effect (ACTIVE vs. PASSIVE) exists, that would be consistent with a general “passive-is-less-risky” bias. Our analysis of participants’ CRRA coefficients across the four treatment conditions shows no evidence of such an effect (Fig. 2). Participants chose similar final lotteries regardless of whether adjustments required active decisions or occurred passively, independent of their initial lottery endowment (SAFE vs. RISKY) and the different adjustment factors (1.5 or 3). Speaking against a “passive-is-less-risky” bias, risk aversion levels were even slightly higher in the passive adjustment treatments, but these differences were not statistically significant at conventional levels (Factor 1.5: RISKY: 1.19 vs. 1.19, p = 0.655; SAFE: 1.65 vs. 1.78, p = 0.481; Factor 3: RISKY: 1.47 vs. 1.54, p = 0.772; SAFE: 2.11 vs. 2.39, p = 0.344; Mann–Whitney U tests). Different types of multivariate regression models controlling for order effects and sample composition differences corroborates these findings (Tables S4, SI), and indicates that the number of adjustment steps—a simple measure of risk aversion—also shows no significant mode-of-choice effect.

The initial lottery endowment (SAFE vs. RISKY) significantly influenced risk-taking behavior, independent of the mode of choice. Participants initially endowed with a safe lottery such that each adjustment step led to more risk-taking exhibited higher levels of risk aversion compared to those initially endowed with the riskiest lottery. This effect was consistent across both adjustment step size factors (Factor 1.5: ACTIVE: 1.65 vs. 1.19, p = 0.006; PASSIVE: 1.78 vs. 1.19, p < 0.001; Factor 3: ACTIVE: 2.11 vs. 1.47, p = 0.019; PASSIVE: 2.39 vs. 1.54, p = 0.002; Mann–Whitney U tests).

There are thus two key results: (1) There is no significant mode-of-choice effect, as average lottery choices are statistically indistinguishable between ACTIVE and PASSIVE conditions, regardless of initial lottery endowments. We thus find no evidence for a general “passive-is-less-risky” bias. (2) The initial lottery endowment significantly affects the average level of risk aversion, with participants initially endowed with a safe lottery choosing less risky lotteries than those initially endowed with the riskiest lottery.

These results, survive a large number of robustness checks. We first asked whether a “passive-is-less-risky” bias occurs for specific rounds with specific lottery payoffs but found no evidence supporting this bias. In one out of nine lotteries (F3-Lowest), participants in the ACTIVE condition even displayed significantly lower levels of risk aversion compared to those in the PASSIVE condition (p = 0.030). In the remaining eight lotteries, mode-of-choice effects were small and not statistically significant (all p > 0.3). Applying a Bonferroni correction for multiple hypotheses testing, none of the coefficients reached the adjusted significance level (SI: Figure S4 and Table S5).

Differences between active and passive decision modes could also manifest in the probability of retaining the originally endowed lottery. However, our results show that the mode of choice does not influence how often participants retain the original lottery. Additionally, even when focusing only on the subset of participants who make at least one adjustment to their initial lottery, the absence of a “passive-is-less-risky” bias remains unchanged (SI: Table S6).

Finally, we demonstrate that within-subject differences between the Dynamic Lottery Adjustment Task (DLAT) and a standard lottery selection task participants complete at the end of the experiment, despite having identical lottery payoffs, are not driven by the choice mode but rather reflect the initial lottery endowments (SI: Figure S6).

In sum, we find little to no evidence for mode-of-choice effects in Experiment 1, a result corroborated by comprehensive robustness checks. Theories suggesting that different modes of choice lead to different lottery selections, due to varying levels of anticipated regret^43^ or a “passive-is-less-risky” bias^34,40,41^, appear unsupported in our setting. This divergence from earlier studies may be due to the hypothetical and context-specific nature of those studies, such as vaccination or investment decisions.^35,36,50^ A second explanation is that earlier vignette studies focus on harmful outcomes relative to a neutral reference point, while our experiment considers regret relative to forgone lottery payoffs. In our context, harm is a loss relative to a higher potential payoff that could have occurred in the same state without additional adjustments. Consequently, realized regret or rejoicing depends on the final lottery outcome. Finally, the apparent null-result in Experiment 1 may be the result of the limited sample size. For the most conservative statistical tests that compare the average level of risk aversion for each participant across decisions and conventional levels of significance ($\alpha = 0.05; \beta =0.80$), we were powered to detect medium to large effect sizes for a each mode-of-choice effect. This would imply a shifts of about 1.29 lottery adjustment steps (out of 10) or a difference in the coefficient of relative risk aversion of about 0.62 due to difference in the choice mode. Yet, the effects we observe are much smaller than that.

### Experiment 2

Experiment 1 shows no general “passive-is-less-risky” bias, suggesting that the willingness to expose oneself to risk are similar across choice modes when all other situational factors are controlled. Given that there are no differences in risk exposure, one key factor that could influence choices is attention cost. Consequently, in Experiment 2, we increased the required attention span. Adjustments were made over a period of 10 days with one adjustment step occurring per day (or requiring an action per day), thereby elevating the potential size of attention costs to implement the next adjustment step in the active condition.

**Fig. 3:**
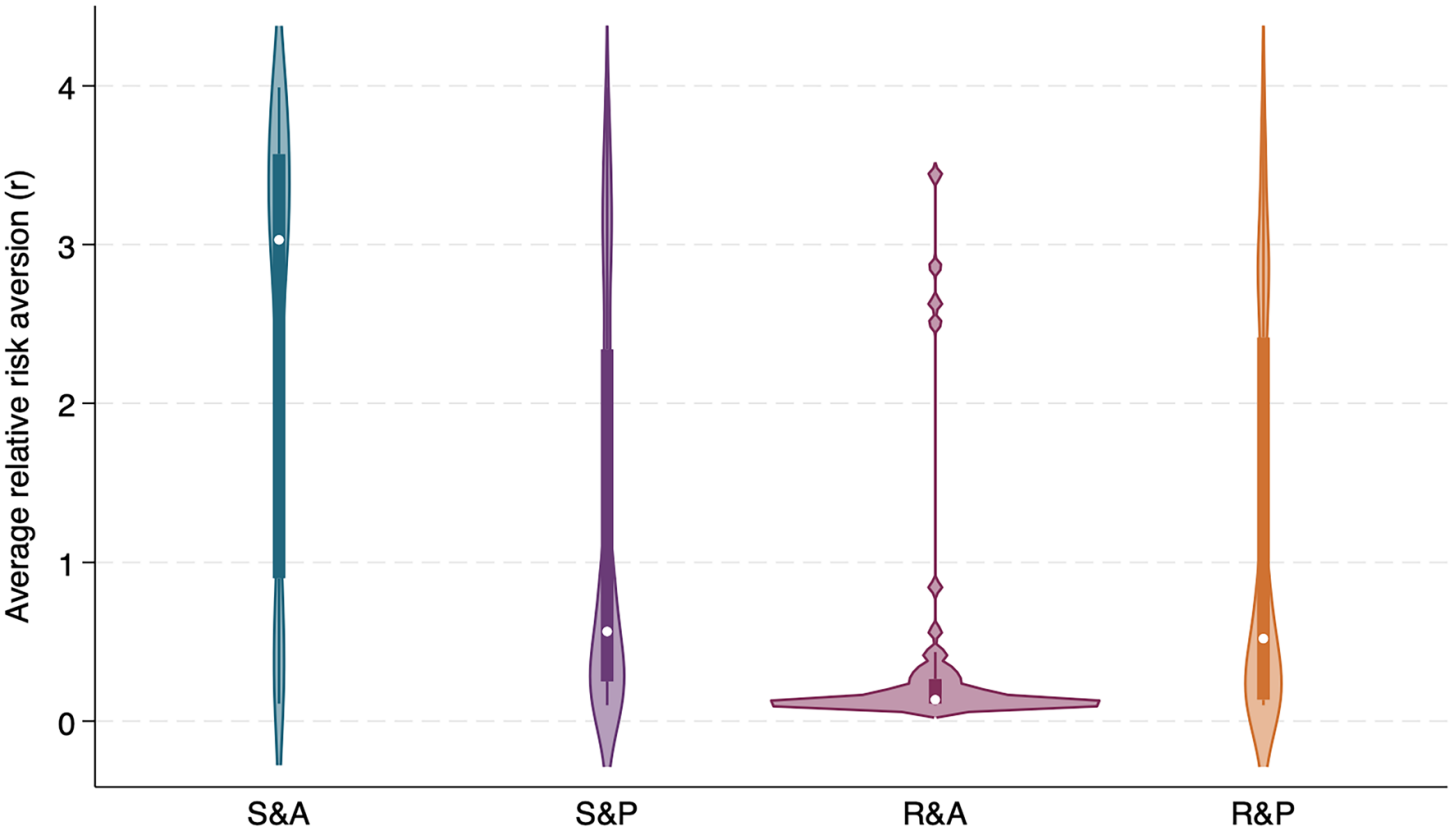
Experiment 2: Risk aversion by treatments. The figure shows violin and box plots of average relative risk aversion coefficients (r) for the full sample, by treatment conditions.

We revisit whether a “passive-is-less-risky” bias emerges, now under high attention cost. Figure 3 illustrates the variation in the CRRA coefficients across the four treatment conditions of Experiment 2, providing strong evidence that the mode of choice (ACTIVE vs. PASSIVE) significantly impacts risk-taking behavior. Consistent with a “passive-is-less-risky” bias, participants initially endowed with a SAFE lottery, where each change increases risk, took higher risks in the PASSIVE condition compared to the ACTIVE condition, as implied by a significantly higher r coefficient in the latter condition (p < 0.001; Mann–Whitney U test). Conversely, inconsistent with a “passive-is-less-risky” bias, participants starting with a RISKY lottery, where each adjustment decreases risk, took significantly less risk in the PASSIVE condition than in the ACTIVE condition (p < 0.001; Mann–Whitney U test). These mode-of-choice effects, though opposite in direction, are similar in absolute size and cancel out when aggregated over the two endowment conditions (p = 0.707; Mann–Whitney U test). Thus, in contrast to Experiment 1, Experiment 2 demonstrates clear mode-of-choice effects, with participants avoiding risks more when avoidance is passive and taking more risks when risk-taking is passive. This opposing pattern supports a mechanism based on attention costs rather than regret avoidance or a “passive-is-less-risky” bias. In accordance with general decision inertia or attention cost models, changes to current risk exposure that require action and attention are less common than those that occur automatically through inaction^51^.

These findings are further supported by regression models controlling for additional individual characteristics or looking at the number of adjustment steps (SI: Table S7). Importantly, participants in the PASSIVE conditions did not simply wait for a similar number of days before stopping adjustments; rather, they adjusted their preferred risk levels according to the initial endowment. In the RISKY condition, implementing this level required 1–2 passive adjustments on average, while in the SAFE condition, it required 8–9 passive adjustments.

As in Experiment 1, initial lottery endowments significantly influenced lottery choices when changes required action (p < 0.001; Mann–Whitney U test). However, this effect disappeared when adjustments occurred automatically (PASSIVE), resulting in similar levels of risk aversion across SAFE and RISKY conditions (p = 0.532; Mann–Whitney U test). The overall significant effect of initial endowment is almost entirely due to the ACTIVE condition (pooled: p < 0.001; Mann–Whitney U test).

**Fig. 4:**
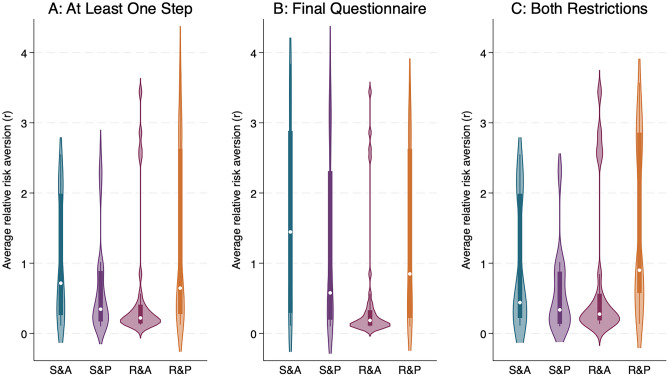
Experiment 2: Risk aversion by sub-samples and treatments. The panels show violin and box plots of average relative risk aversion coefficients (r) by treatment conditions. Panel (**A**) restricts the sample to those who took at least one adjustment step. Panel (**B**) restricts the sample to those who completed the final online questionnaire. Panel (**C**) combines both sample restrictions.

Given the multi-day duration of the experiment, it is crucial to determine whether some findings might be driven by participants who disengage early or late in the experiment. To assess the robustness of the mode-of-choice effects, we repeated the analysis for several subsamples, focusing on participants who displayed a minimum level of attentiveness by making at least one change to the lottery payoffs or by completing the final questionnaire (Fig. 4). In these more attentive subsamples, we continue to find evidence for mode-of-choice effects.

Panel A shows results for 138 participants (65%) who implemented at least one adjustment step. For participants in the RISKY condition, the mode-of-choice effect is similar in size and direction to that in the full sample (p < 0.001; Mann–Whitney U test). In the SAFE condition, the mode-of-choice effect is smaller and statistically insignificant (p = 0.168; Mann–Whitney U test). Panel B restricts the sample to the 115 participants who logged in after 12 days to complete the final questionnaire. Again, in the RISKY condition, we find strong evidence of a mode-of-choice effect (p < 0.001; Mann–Whitney U test), while in the SAFE condition, the effect remains smaller and not statistically significant (p = 0.251; Mann–Whitney U test). Panel C, which combines both sample restrictions and includes 86 participants, yields similar results: a significant mode-of-choice effect in the RISKY condition (p = 0.004; Mann–Whitney U test) and an insignificant effect in the SAFE condition (p = 0.251; Mann–Whitney U test). In the SI: Table S8 (Section C), we show that there is no evidence for treatment specific attrition and that retention of the original lottery is lower in the ACTIVE than in the PASSIVE conditions.

Overall, these robustness checks shows that mode-of-choice effects persist, particularly in the RISKY condition, where adjustment steps reduce risk exposure. Participants are more likely to display risk-averse behavior when adjustments toward safer lottery payoffs occur automatically rather than requiring action. In the SAFE condition, where adjustments lead to more risk-taking, mode-of-choice effects do not reach statistical significance. Jointly this contradicts a general “passive-is-less-risky” bias, which would predict the opposite i.e. more risk-taking in PASSIVE than in the ACTIVE conditions.

## Discussion and conclusion

Our study investigates whether different modes of choice, specifically active versus passive decision-making, systematically affect risk-taking behavior, as claimed by previous research. To explore this, we developed the DLAT, a stepwise adjustment task that allows for the study of active and passive risk-taking both when action (inaction) lead to more or less risk exposure. The DLAT can easily be amended to other research questions, parameterisations or durations. To understand if there is a “passive-is-less-risky” bias, we conducted two experiments to test for mode-of-choice effects with and without attention costs. Experiment 1, a standard laboratory experiment, involved minimal attention costs for participants. In contrast, Experiment 2, conducted online over 10 days, introduced significant attention and effort costs, better reflecting some natural decision environments like they occur in financial decision making.

In both experiments, initial lottery endowments influenced participants’ subsequent choices, consistent with prior findings on status-quo bias^37^. However, our main findings regarding the mode of choice depend on the presence of attention costs. Experiment 1 showed no statistically significant mode-of-choice effects, whereas Experiment 2 revealed pronounced effects. In neither case do we find that risk-taking is consistently increased when choices are made through inaction rather than action. This speaks against a general “passive-is-less-risky” bias. Instead, when attention costs are high, we observe more risk-taking when inaction promotes risk-taking, and vice versa.

The fact that mode-of-choice effects only appear in the presence of attention costs hints at one potential explanation for why abstract experimental procedures that do rely on active lottery selection and do not account for important contextual features often have low predictive power for real-world behavior^52–55^. In many experimental settings, effort, attention, and opportunity costs are negligible because participants have already committed the time and effort to participate. Outside of tightly controlled laboratory experiments, these factors significantly impact decision-making and can strongly influence risk-taking behavior depending on the mode of choice. Our results suggest that to develop risk-elicitation methods with high predictive power beyond the lab, it is crucial to consider contextual features and the interaction between decision mode and attention costs.

Our study is not without its own limitations, however. One reason for the apparent null result in Experiment 1 might be a lack of statistical power. Thus, the true effect might be too small to be detected given our sample size. As outlined in the methods’ section, we were powered to detect effect sizes corresponding to a difference between the control and treatment groups of about 1.05 to 1.29 lottery adjustment steps, depending on wether we account for the second treatment dimension of the starting point. The observed differences in Experiment 1 were much lower (e.g., 0.13 steps for the lotteries with a safe initial lottery endowment). If this difference was representative for the true effect size, a much larger sample would be required to identify the effect at sufficient power. The flip-side of this would be that the effect, even if statistically significant, would not be meaningful. Moreover, in both of our experiments, we draw on a convenience sample of German university students. This sample is relatively young, highly educated, and likely comes from a very homogeneous cultural background. It has been shown that age and culture both affect the level of risk aversion as measured by standard economic tasks^56–58^. Yet, previous research also indicates that the fundamental patterns and treatment effects observed for risk and uncertainty attitudes appear to be broadly robust to cultural factors, despite the observed level shifts^18,59^. We opted for a homogeneous convenience sample in a tightly controlled laboratory setting in this study as this setting is often advantageous for initially detecting and identifying novel phenomena with relatively little noise, and thus with smaller sample size requirements. However, we strongly encourage further studies to test the generalisability of our findings across cultures and age groups using a more diverse and representative subject pool.

In conclusion, both active and passive risk-taking occur across many domains of decision-making. Previous research has suggested a general tendency to perceive passive risks as less risky, leading to greater acceptance of such risks. However, this research has primarily focused on specific scenarios and hypothetical vignette or survey designs. Here, we offer a complementary behavioral measure of passive risk-taking. In a tightly controlled lab setting, we find no evidence that the mode of decision-making affects the amount of risk taken, ruling out the possibility that passive risks are perceived differently from active risks at a general level. Instead, our results suggest that attention costs drive differences between active and passive risk-taking, with effects differing based on whether inaction leads to more or less risky behavior. This indicates that passive risk-taking and passive risk-avoidance are both specific cases of general inaction phenomena i.e. a failure to take action in face of attention or more general decision cost.

## Data Availability

A replication package, including instructions in German and English language, raw data, and data analysis files is available in an OSF repository at: https://osf.io/uy9nx/.

## Declarations

### Complete reporting

The authors declare to have reported on all experimental treatments that were conducted and that all measured variables are disclosed in the manuscript or the Supplementary Material. They have not conducted any other study on the specific research question of this article.
